# Foldable and High Sulfur Loading 3D Carbon Electrode for High-performance Li-S Battery Application

**DOI:** 10.1038/srep33871

**Published:** 2016-09-28

**Authors:** Na He, Lei Zhong, Min Xiao, Shuanjin Wang, Dongmei Han, Yuezhong Meng

**Affiliations:** 1 The Key Laboratory of Low-carbon Chemistry & Energy Conservation of Guangdong Province/State Key Laboratory of Optoelectronic Materials and Technologies, School of Materials Science and Engineering, Sun Yat-sen University, Guangzhou 510275, P. R. China; 2 Sino-French Institute of Nuclear Engineering and Technology, Sun Yat-sen University, Zhuhai 519082, P. R. China

## Abstract

Sulfur is a promising cathode material with a high theoretical capacity of 1672 mAh g^−1^, however, the practical energy density of Li-S battery is far away from such promising due to its low active material utilization and low sulfur loading. Moreover, the challenges of the low electrical conductivity of sulfur and the high solubility of polysulfide intermediates still hinder its practical application. Here, we report a kind of free-standing and foldable cathodes consisting of 3D activated carbon fiber matrix and sulfur cathode. The 3D activated carbon fiber matrix (ACFC) has continuous conductive framework and sufficient internal space to provide a long-distance and continuous high-speed electron pathway. It also gives a very larger internal space and tortuous cathode region to ACFC accommodate a highly sulfur loading and keeps polysulfide within the cathode. The unique structured 3D foldable sulfur cathode using a foldable ACFC as matrix delivers a reversible capacity of about 979 mAh g^−1^ at 0.2C, a capacity retention of 98% after 100 cycles, and 0.02% capacity attenuation per cycle. Even at an areal capacity of 6 mAh cm^−2^, which is 2 times higher than the values of Li-ion battery, it still maintains an excellent rate capability and cycling performance.

Lithium–sulfur (Li-S) batteries are amongst the most promising next-generation battery technologies due to their high theoretical energy density, environmental friendliness and abundant in nature^1^,^2^. Despite these considerable advantages, a series of obstacles still hinder the practical application of this attractive cathode material^3^. The main challenges include the poor electrical conductivity of elemental sulfur and its discharge product Li_2_S, which results in limited active material utilization and low rate capability^4^,^5^; the volumetric expansion of sulfur upon lithiation and the high solubility of the intermediate products of lithium polysulfides (Li_2_S_x_, 4 ≤ x ≤ 8) in the organic electrolyte solution, resulting in irreversible active sulfur loss, rapid capacity decay and low Coulombic efficiency during cycles^6^,^7^,^8^,^9^.

To overcome these drawbacks of the sulfur cathode, many approaches have been explored to improve the electronic conductivity of sulfur and prevent the dissolution of polysulfides into the electrolyte, including preparation of hierarchical porous carbon–sulfur composites as sulfur host^10^,^11^,^12^,^13^,^14^,^15^,^16^. And it is believed that encapsulating the sulfur in carbon improves its utility as an active mass and avoids the diffusion of the Li_2_S_n_ species to the electrolyte solution, thus it reduces the shuttle phenomena that limit the capacity of sulfur cathodes^17^,^18^.

For example, Zhang *et al*.^10^ prepared a sulfur–carbon sphere composite by encapsulating sulfur into micropores of carbon spheres. It is demonstrated from galvanostatic discharge–charge process, the composite with 42 wt% sulfur presents a long electrochemical stability up to 500 cycles, retained a capacity of 650 mAh g^−1^ with current density of 400 mA g^−1^. Wang’s group^15^ reported the multi-shelled hollow carbon nanospheres show a high specific capacity of 1350 mA h g^−1^ and excellent capacity retention (92% for 200 cycles). Elazari *et al*.^17^ reported a binder-free carbon-sulfur cathodes for Li-S batteries by impregnation of microporous activated carbon fibers with elemental melted sulfur (ACF–S), the monolithic carbon-cloth/sulfur electrodes have demonstrated the maximum discharge capacity of 1050 mAh g^−1^ sulfur. The utilization of the sulfur is effectively improved but there is further research to do with the stability of cycle performance.

In addition, the researchers have paid more attention on the structure of the electrode especially the different matrices^18^,^19^,^20^,^21^,^22^. As we knew that the traditional current collector is Al foil, if it were replaced with three-dimensional matrix, the contacting area of the active material and the supports would be much greater. Furthermore it would be effectively buffer the volume expansion during the charge-discharge process.

In this work, we report here with a novel, simple, and very promising method for a foldable and highly sulfur loading electrode for Li-S batteries. A commercial foldable activated carbon fiber cloth (ACFC) was used as cathode matrix. ACFC–S cathodes were simply prepared by blade coating method. ACFC has high electric conductivity with continuous long-distance conductive structures and plentiful internal space to accommodate a large amount of active sulfur. What’s more, ACFC has a surface area of 471 m^2^ g^−1^ and plenty of micropores, which is expected to keep the polysulfide within the cathode.

## Results

### Preparation and characterizations of composite cathodes

The active material slurry was prepared by mixing 70 wt% sublimed sulfur, 20 wt% super P as a conducting agent, and 10 wt% PVDF as a binder in NMP. The commercial activated carbon fiber cloth was treated and modified, and then used as cathode matrix (ACFC). Composite cathodes were prepared by a simple blade coating method to permeate slurry into matrix. The active material was well dispersed into the interspaces, and was uniformly coated onto the carbon fibers. As contrast, the commercial carbon fiber paper (CFP) and traditional Al foil were used as matrices to prepare cathodes in the same way. Composite cathodes with ACFC, CFP and Al foil as matrix were named ACFC–S cathode, CFP–S cathode and Al-S cathode respectively. ACFC–S cathodes and CFP–S cathodes were directly used as free-standing electrode without Al foil. Sulfur loading of ACFC–S cathode is 3–4.5 mg cm^−2^, and about 1.5 mg cm^−2^ of CFP–S cathode, about 1 mg cm^−2^ of Al–S cathode.

Different with planar Al foil, ACFC have 3D structure and netlike electron pathway. ACFC is composed of intertexture carbon fibers which have high conductivity and solvent taken-up ability that will be demonstrated below. Table S1 shows the elemental composition of ACFC and CFP. It can be seen that ACFC contains 79.18% of C and 4.78% of N, while N content in CFP is nearly as 3%. The very high carbon content endows high electron conductivity. It was found that the presence of nitrogen dopants in ACFC can enhance the electronic properties, and also facilitates polysulfide binding via chemical interactions^23^,^24^. Figure S1a,b show the scanning electron microscopy (SEM) images of ACFC. The diameter of carbon fibers is about 5 μm. It can also be seen that there are some large interspaces which is important for ACFC to accommodate more sulfur inside and increase their utilization. In this sense, the ACFC serves as a reservoir for liquid electrolyte and thus ensures good electrolyte immersion. The nitrogen (N_2_) adsorption and desorption isotherms, DFT pore-size distribution curves of ACFC are shown in Fig. S2a,b. ACFC has high microporosity according to IUPAC Type I isotherms with a surface area of 471 m^2^ g^−1^and a total pore volume of 0.249 cm^3^ g^−1^ (detected pore size smaller than 207 nm). The ACFC have also good flexibility, which can guarantee a foldable ACFC–S cathode electrode. Figure S1c,d show photographs of the ACFC–S cathode. The ACFC–S cathode can be easily folded without brittle fracture, and the active material can be firmly adhered inside ACFC even after numerous folding.

### Morphology and dispersion of sulfur within composite cathode

**Figure 1:**
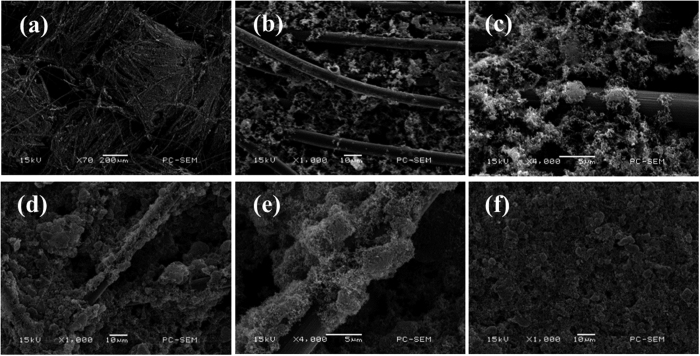
SEM images of (**a**–**c**) ACFC–S cathodes, (**d**,**e**) CFP–S cathodes and (**f**) Al–S cathodes.

**Figure 2:**
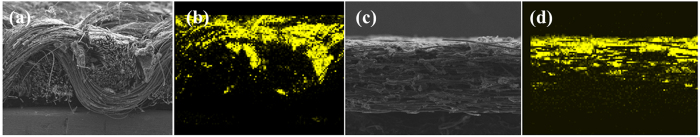
SEM images and corresponding sulfur mapping images of cross-sectional (**a**,**b**) ACFC–S cathodes and (**c**,**d**) CFP–S cathodes.

The morphology and dispersion of sulfur within ACFC–S and CFP–S cathodes were observed by SEM and the corresponding sulfur mapping images are shown in Figs 1a–e and 2a–d respectively. Unlike Al–S cathodes (Fig. 1f), smaller active material particles are filled in the interspaces or adhere onto carbon fibers in ACFC or CFP. The larger contact area between sulfur and carbon fibers can play a role as inner electron pathway as well as current collector. The thin and uniform active material layer on the carbon fiber ensures the good contact of sulfur particles and conductive substrate, and serves as a short-cut electronic pathway from the bulk sulfur to ACFC matrix. It can also be found that there are still some voids within ACFC–S after accommodation of sulfur within ACFC, which favors the electrolyte immersion in electrolyte and the transportation of Li^+^ transport. The sulfur mapping images of cross-sectional ACFC–S cathodes (Fig. 2a) indicates that the active material was successfully permeated into ACFC. The CFP–S cathode (Fig. 2d) is similar to ACFC cathode. Moreover, it can be seen that a gradient distribution of sulfur within the cross-section existed, and less sulfur is found far away from the sulfur coated surface of ACFC matrix.

### Redistribution of sulfur within ACFC-S cathodes after cycles

**Figure 3:**
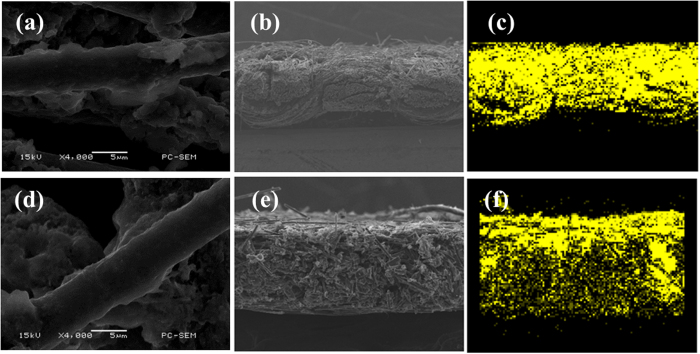
SEM images and corresponding sulfur mapping images of cycled (**a**) surface (**b**,**c**) cross-sectional ACFC-S cathodes, and cycled (**d**) surface (**e**,**f**) cross-sectional CFP-S cathodes.

The cycled ACFC–S cathodes and CFP–S cathodes were carefully investigated after 70^th^ charge. Figure 3a,d show that the active sulfur particles formed random flakes and blocks, which covered on the surfaces of ACFC fibers. The dispersion of sulfur in ACFC or CFP becomes more uniform, and the surface area of uncovered ACFC fibers decreases greatly. Consequently, the adhesion between sulfur and ACFC matrix becomes much better compared with uncycled one. Figure 3c,f indicate that the sulfur was totally permeated into the inside of ACFC and CFP and dispersed more homogeneously after cycles, especially for ACFC–S cathode. Sulfur is herewith subjected to a solid_(sulfur)_–liquid_(polysulfide)_–solid_(sulfide)_ phase transition during the cycling. The electrolyte containing soluble polysulfide can be trapped and adsorbed by the tortuous cathode matrix, and the polysulfide can be electrochemically deposited onto ACFC fibers, which then induce the redistribution of sulfur after cycling. The agglomeration of sulfur can be greatly reduced due to the redistribution, which leading to a more uniform cathode structure and an improved effectively utilization of sulfur.

### Electrochemical performance

**Figure 4:**
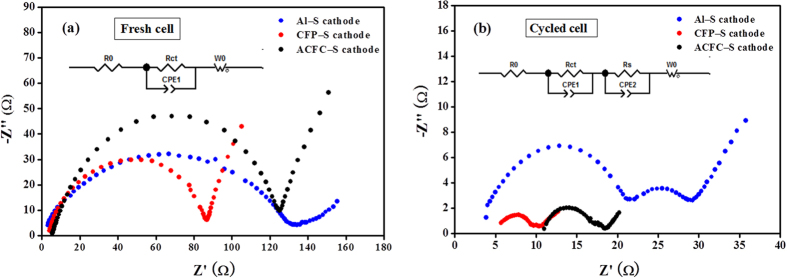
Nyquist plots of (**a**) fresh cathodes and (**b**) cathodes after 10 cycles.

To account for the enhanced electrochemical performance of the 3D structured composite cathodes, the electrochemical impedance spectroscopy (EIS) of coin cells with three different cathodes was performed (shown in Fig. 4a,b). A typical Nyquist plots of Li-S batteries is normally consisted of semicircles in the high and middle frequency region and an inclined in low frequency region. The first semicircle at high and middle frequency is attributed to the charge transfer of sulfur intermediates, and the following semicircle is due to the formation and dissolution of S_8_ and Li_2_S^25^. As shown in Fig. 4a, there is only one semicircle in Nyquist plots for the batteries before cycling, which represents charge transfer resistances. Compared to conventional Al–S cathodes, the semicircles of both ACFC–S cathodes and CFP–S cathodes show smaller diameter in the first semicircle, implying that the interfacial and charge-transfer resistances decrease effectively with the 3D structure design for the as-assembled batteries. A Li_2_S passivation layer is proposed to be formed and coated onto the surface of matrix or active material after cycles, thus an additional semicircle in the middle frequency region of the Nyquist plot appears. Furthermore, the semicircle diameters of ACFC–S cathodes and CFP-S cathodes are extremely small, indicating very little inactive Li_2_S were formed and highly sulfur utilization because of the large reaction area of the electrode. It should be noted that the charge transfer resistances of cycled cathodes decrease obviously compared with fresh cathodes for both ACFC–S and CFP–S cathodes, because of the more uniform distribution of sulfur within cathode after cycles. Accordingly, the impedance of both cathodes decreases. This is even more obvious for ACFC–S and CFP–S cathodes, because there are large amount of interior space inside the matrices and the carbon fibers wrapped with sulfur during cycles. Therefore, the impedance of both ACFC-S and CFP-S cathodes decrease much obviously compared with Al-S cathode. These results are consistent with those of SEM images.

**Figure 5:**
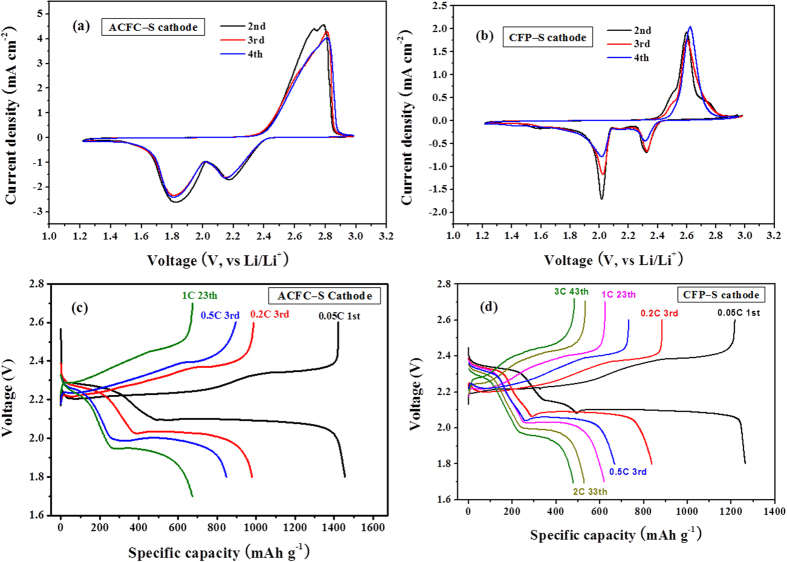
Cyclic voltammograms of (**a**) ACFC–S cathodes at scan rate 0.05 mV/s, (**b**) CFP–S cathodes at scan rate 0.1 mV/s. and charge-discharge profiles of (**c**) ACFC–S cathodes, (**d**) CFP–S cathodes at different current density.

The electrochemical behavior and reversibility of both ACFC–S and CFP–S cathodes were investigated by cyclic voltammetry (CV) measurements and charge/discharge voltage profiles. All experimental curves are shown in Fig. 5a–d. There are two cathode peaks and a broad anode peak in the CV curves for composite cathodes. The broad anode peak can be considered as two overlapped peaks. The two cathode peaks, starting at 2.4 V and 2.05 V respectively, correspond to the two-step reduction reaction from elemental sulfur (cyclic S_8_) to soluble Li_2_S_x_ (40 ≤ x < 8) and then to insoluble Li_2_S_2_/Li_2_S^20^. In the subsequent anodic scan, a broad anode peak starting 2.4 V corresponds to the oxidation of reduction products. The potentials corresponding to the reduction peaks and oxidation peak of ACFC–S cathode shift to right slightly because of the polarization resulted from high current density and high sulfur loading. Moreover, the shift of the potential in cathode and anode peaks during cycling is not obvious, especially for ACFC–S cathodes which still show good reproducibility of current, indicating good electrochemical reversibility.

Figure 5c,d present the charge-discharge voltage profiles of coin cells using ACFC–S and CFP–S cathodes at different current density, respectively. ACFC–S cathodes deliver a high initial discharge capacity of above 1400 mAh g^−1^, and nearly 1300 mAh g^−1^ for CFP–S cathodes at a low current density 0.05C, while the initial discharge capacity of conventional Al–S cathodes is less than 900 mAh g^−1^. For the cycles at higher current density, both ACFC–S and CFP–S cathodes still exhibit high capacity, for example, 979 mAh g^−1^ at 0.2C, 848 mAh g^−1^ at 0.5C for ACFC–S cathodes, and 839 mAh g^−1^ at 0.2C, 668 mAh g^−1^ at 0.5C for CFP–S cathodes. Two voltage plateaus at 2.3 V and 2.1 V are observed in turn during discharge processes, which are in accordance with typical Li-S batteries. However, the over potential of coin cells increases with the increase of current density. This corresponds to a larger voltage hysteresis between the charge and discharge curves (ΔE), especially for ACFC–S cathodes. Because of the high sulfur loading of ACFC–S cathode, the current density at 0.2C is higher than 1.2 mAh cm^−2^, which is 5 times of Al-S cathode in a coin cell. At further higher current density, the polarization occurred not only onto the cathode, but also the anode and even the electrolyte. These factors resulted in higher over-potential on ACFC–S cathodes. For the electrical appliances, a higher current density plays an important role, which could offset some loss resulted from the over-potential.

**Figure 6:**
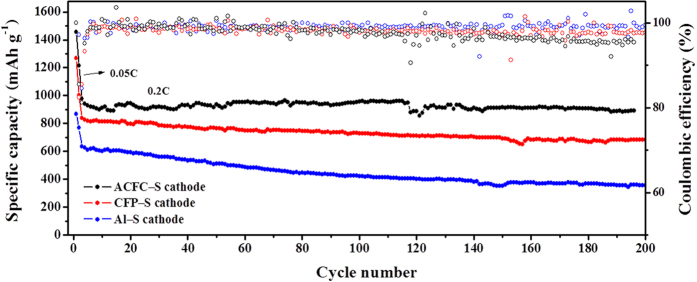
Cycling performance at current density: 0.05 C of the first two cycles and 0.2 C of the follow cycles.

The long-term cycling stability of the cells using different cathodes at 0.2C (Fig. 6) and 0.5C (Fig. S3) were tested at the voltage of 1.8–2.6 V. The reversible discharge capacity after activation cycles is about 979 mAh g^−1^, 839 mAh g^−1^, 634 mAh·g^−1^ for ACFC–S, CFP–S, and Al–S, in their turns. The capacity of ACFC–S cathode increases with the cycles, and their capacity retention after 100 cycles is 98%, and 0.02% capacity attenuation per cycle, which demonstrates an excellent cycling stability. The capacity retentions of CFP–S cathode and Al–S cathode are 87% and 67% after 100 cycles respectively. The ACFC–S cathodes show excellent cycling stability according to CFP–S cathode and Al-S cathode. The rate performance of ACFC–S cathode and CFP–S cathode were tested at the voltage of 1.8–2.6 V (below 1C) or/and 1.7–2.7 V (1C and higher)^26^, which evaluated at different current densities from 0.2C to 1C and 0.2C to 3C respectively, as shown in Fig. S4. The discharge capacity decreases slightly with increasing current density, especially for ACFC–S cathodes. When the current density finally changes back to 0.2C after dozens of cycles, the ACFC–S cathodes can almost recover to its original capacity. The good cycling and rate performance of ACFC–S cathodes can be ascribed to the 3D structure of the composite cathode. The said structure provides fast electronic transport promoted by 3D conductive network, high Li^+^ accessibility. This is because of the good electrolyte immersion and channels among carbon fibers, good accommodation and highly effective utilization of active material resulting from the interspace of ACFC, and the cage effect limiting lithium polysulfide to diffuse out of cathode region.

## Discussion

Based on the above experimental results, it can be concluded that the electrochemical performance of the composite cathodes based on 3D carbon fiber framework is much better than that of the conventional Al–S cathode. Particularly, the ACFC–S cathode gives an excellent performance with high reversible capacity, extremely high capacity retention, as well as high rate capability. It is believed that not only the conductive nature and higher content of N (4.78%), but also plentiful micropores of ACFC account for such superior performance. Moreover, the 3D woven structure can keep well the filled active materials inside the cathode, and the content of PVDF adhesive can be reduced to a very low level.

**Figure 7:**
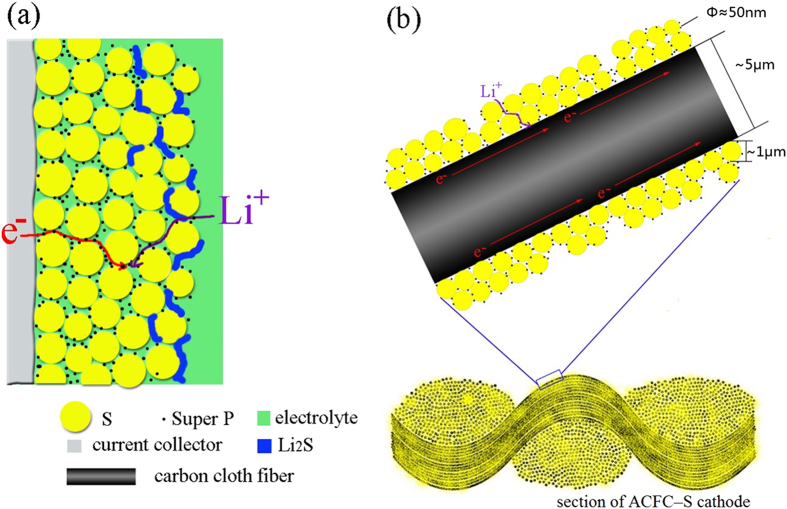
Schematic of electron pathway of (**a**) conventional sulfur cathode, (**b**) ACFC–S cathode with 3D conductive framework.

In order to understand better and discuss well the superior electrochemical performance for the 3D structure cathode, a schematic working image of this cathode is shown in Fig. 7. It is clear that the predominant factor is the continuous conductive framework 3D cathode. As shown in Fig. 7a, the aluminum foil of conventional Al–S cathode has a limited contact area with active material, so it only acts as current collector and leads it to external circuit. The electrical conductivity of the cathode is therefore not satisfied. It is believed that there is only point-to-point contact between spherical carbon black and sulfur particles, which cannot serve as a continuously conductive path. In this case, an electron transfer from the current collector to active sulfur suffers from a long distance and un-direct path way that will certainly increases the inner electron resistance. For the composite cathodes with 3D conductive framework, however, the continuous carbon fibers act as high-speed electron pathway. Active material can contact firmly each other, in which continuous carbon fiber and a small amount of spherical carbon black built up a hierarchical “dot-line” conductive framework. Undoubtedly, the jointly “dot-line” structure can effectively improve the conductivity of the whole cathode. Secondly, the active material is encapsulated within inner voids of ACFC woven structure that can effectively prevent polysulfides to diffuse out of the cathode. Finally, the internal space and high mechanical properties can tolerate and buffer the volumetric change of the active material during cell cycling. It should be particularly emphasized that ACFC can also effectively adsorb electrolyte because of its plentiful micropores, thus the polysulfides are limited within the cathode region and without diffusing into electrolyte.

In order to increase the sulfur loading level, we coated the active materials on either side of the ACFC cathodes. In this case, the loading of sulfur can be easily increased up to 6–7.2 mg cm^−2^ (denoted as DL-ACFC–S cathode). The charge–discharge measurements of DL-ACFC–S cathode were carried out at the current density of 0.1C (about 1.15 mAh cm^−2^) and the profiles were shown in Figs S5 and 8. The reversible capacity is 788 mAh·g^−1^ at 0.1C after activation cycles. It can be seen that the capacity is further improved during cycles; presumably, this is due to the longer activation process of the larger amount of sulfur used. In this sense, it can be concluded that the electrochemical performance was found even better after increasing sulfur loading.

The areal capacity of lithium-sulfur battery is calculated based on to sulfur loading of 7 mg cm^−2^ and specific capacity of 850 mAh g^−1^ in Equation (1):


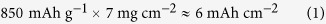


While the sulfur loading of most lithium sulfur battery reported in the literature is less than 2 mg cm^−2^, and the average specific capacity is about 1000 mAh g^−1^, which means that the areal capacity is less than the result of Equation (2):


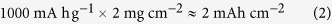


Moreover, the areal capacity of commercial Li-ion battery is about 3 mAh cm^−2^. It can be concluded that the value of 6 mAh cm^−2^ of ACFC-S cathodes is 3 times higher than the values of Li-S battery with conventional configuration reported in the literature^27^,^28^,^29^, and 2 times higher than the values of Li-ion battery. It is expected that the electrochemical performance of such novel 3D cathode could be further enhanced with further increasing sulfur loading technically. What’s more, the ACFC–S cathode can be folded freely without any brittle fracture, which meets the requirement of both soft-package battery and cylindrical battery, leading to a great potential for commercial applications.

## Methods

### Preparation of ACFC-S, CFP-S, and conventional Al-S cathode

The active material slurry was prepared by mixing sublimed sulfur (Aladdin), super P (TIMCAL), and PVDF (Arkema) in a mass ratio of 7:2:1, and then stirred with NMP. The mixture was fixed in a planetary ball-mall machine (QM-3SP04, Nanjing) and agitated at 200 rpm in air for 2 h. The activated carbon fiber cloth (LISO, Shanghai) was washed with DI water by sonication, then dried at 80 °C. The composite cathode films were formed by a doctor blade deposition of obtained slurry into ACFC without gap between the matrix and blade. Sulfur loading can be adjusted by controlling the slurry and coating operation. The ACFC–S cathode was then dried at 60 °C for 3 h in a convection oven and 60 °C in a vacuum oven for 24 h. CFP-S cathode was made in the same way using carbon fiber paper (Toray) as matrix. Al–S cathode was prepared by a conventional method with a coating thickness about 100 μm.

### Material characterization

Elemental analyses (EA) were performed on Elementar Vario EL Cube elemental analyzer to determine the sulfur content in ACFC. Nitrogen adsorption−desorption isotherms and pore size distribution of ACFC were characterized by accelerated surface area and porosimetry system (Micromeritics, ASAP 2010). The morphology and dispersion of sulfur of composite cathodes were observed with scanning electron microscopes (SEM, JEOL, JSM-6380LA and SEM, JEOL, JSM-6330F) equipped with energy dispersive X-ray spectrometer (EDS).

### Cell assembling and electrochemical measurements

CR2025-type coin cells were used as the testing cells. Cathodes were punched into φ14 mm, and directly used as free-standing cathodes. Lithium foil were used as anodes, Cellgard 2500 were used as separator, and 1 M LiTFSI in a 1:1 volume of DME:DOL and 2 wt% LiNO_3_ were used as electrolyte. The amount of electrolyte was about 60, 55, 50 μL for ACFC–S cathode, CFP–S cathode, Al–S cathode respectively. Cyclic voltammetry (CV) and electrochemical impedance spectroscopy (EIS) were carried out using a Solartron 1255B frequency response analyzer coupled with a Solartron 1287 electrochemical interface at a scan rate of 0.1 or 0.05 mV s^−1^ from 1.2 to 3.0 V versus Li^+^/Li and in the frequency range from 100 MHz to 0.01 Hz with an applied voltage amplitude of 5 mV, respectively. Charge–discharge measurements were carried out in a voltage window of 1.8–2.6 V or 1.7–2.7 V at different current densities and at room temperature by a NEWARE battery tester.

## Additional Information

**How to cite this article**: He, N. *et al*. Foldable and High Sulfur Loading 3D Carbon Electrode for High-performance Li-S Battery Application. *Sci. Rep.* **6**, 33871; doi: 10.1038/srep33871 (2016).

## Supplementary Material

Supplementary Information

## Figures and Tables

**Figure 8:**
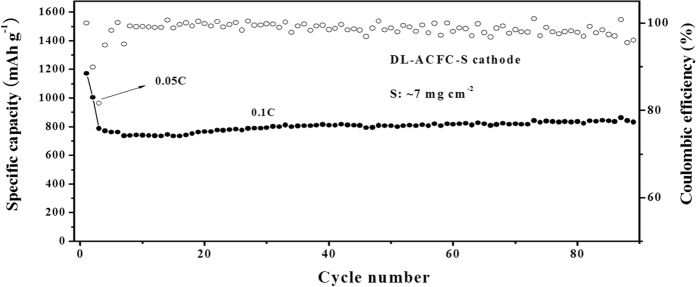
Cycling performance at current density: 0.05 C of the first two cycles and 0.1 C of the follow cycles.
