# Supplementary material for: Foldable and High Sulfur Loading 3D Carbon Electrode for High-performance Li-S Battery Application
Source: Sci Rep. 2016 Sep 28;6:33871. doi: 10.1038/srep33871 (PMC5039404; doi:10.1038/srep33871)
Supplement: Supplementary Information [file srep33871-s1.doc]

# Foldable and High Sulfur Loading 3D Carbon Electrode for High-performance Li-S Battery Application

Na He ^2,+^, Lei Zhong ^2,+^, Min Xiao ^2^, Shuanjin Wang ^2^, Dongmei Han ^1^,*, Yuezhong Meng ^2^,*

^1^ *Sino-French Institute of Nuclear Engineering and Technology, Sun Yat-sen University, Zhuhai 519082, P. R. China;*

^2^ *The Key Laboratory of Low-carbon Chemistry & Energy Conservation of Guangdong Province/State Key Laboratory of Optoelectronic Materials and Technologies, School of Materials Science and Engineering, Sun Yat-sen University, Guangzhou 510275, P. R. China.*

*E-mail:* [*mengyzh@mail.sysu.edu.cn*](mailto:mengyzh@mail.sysu.edu.cn) *(Y. Z. Meng), and* [*handongm@mail.sysu.edu.cn*](mailto:handongm@mail.sysu.edu.cn) *(D. M. Han); Fax: +86 20 84114113; Tel: +86 20 84114113.* ^+^: *Who contribute equally to this work.*

Fig. S1


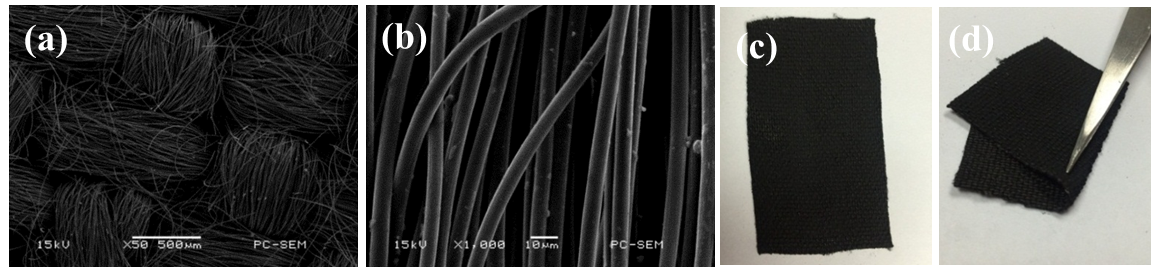


Fig. S1 SEM image of (a, b) activated carbon fiber cloth and (c, d) photograph of ACFC–S cathode.

Fig. S2

Fig. S2 (a) Nitrogen adsorption–desorption isotherms and (b) DFT pore-size distribution curves of ACFC.

Fig. S3

Fig. S3 Cycling performance of composite cathodes at current density: 0.05C of the first two cycles, and 0.5C of the follow cycles.

Fig. S4

Fig. S4 Rate capacity of different cathodes at different current density.

Fig. S5

Fig. S5 Charge-discharge profile of high-loading sulfur DL-ACFC-S cathode in 3rd cycle.

Table S1

Table S1 Elemental composition of ACFC and CFP.

|  | C (%) | H (%) | N (%) | C/N ratio | C/H ratio |
| --- | --- | --- | --- | --- | --- |
| ACFC | 79.18 | 2.05 | 4.78 | 16.56 | 38.70 |
| CFP | 90.08 | 2.78 | 2.91 | 31.01 | 32.43 |
